# Surveillance and treatment of primary hepatocellular carcinoma (aka. STOP HCC): protocol for a prospective cohort study of high-risk patients for HCC using GALAD-score

**DOI:** 10.1186/s12885-023-11167-9

**Published:** 2023-09-18

**Authors:** Thai Ngoc Truong, Trang Ngoc Doan Pham, Long Bao Hoang, Van Thi Nguyen, Hang Viet Dao, Diem Vu Bich Dao, Saleh Alessy, Hien Ba Pham, Thuy Thi Thu Pham, Linh Duc Duy Nguyen, Khue Nguyen, Faisal Abaalkhail, Mohammed Manal, Mohammad Mawardi, May AlZahrani, Khalid Alswat, Hamdan Alghamdi, Faisal M. Sanai, Mohammed Amir Siddiqui, Nam Hai Nguyen, Dhananjay Vaidya, Hai Thanh Phan, Philip J. Johnson, Saleh A. Alqahtani, Doan Y Dao

**Affiliations:** 1 Department of Internal Medicine, Hanoi Medical University, Hanoi, Vietnam; 2 School of Public Health, the University of Illinois in Chicago, Chicago, IL USA; 3 Institute of Gastroenterology and Hepatology, Hanoi, Vietnam; 4 Vietnam Viral Hepatitis Alliance, Reston, VA USA; 5 College of Health Sciences, the Saudi Electronic University, Jeddah, Saudi Arabia; 6 Dong Da General Hospital, Hanoi, Vietnam; 7 Medic Medical Center in Ho Chi Minh, Ho Chi Minh City, Vietnam; 8 Medic Medical Center in Rach Gia, Rach Gia City, Kien Giang Province Vietnam; 9 Medic Medical Center in Ca Mau, Ca Mau City, Vietnam; 10 Department of Medicine, Section of Gastroenterology, King Faisal Specialist Hospital and Research Center, Riyadh, Saudi Arabia; 11 College of Medicine, Al-Faisal University, Riyadh, Saudi Arabia; 12 Department of Internal Medicine, King Faisal Specialist Hospital & Research Center, Riyadh, Saudi Arabia; 13 Department of Internal Medicine, King Faisal Specialist Hospital & Research Center, Jeddah, Saudi Arabia; 14 Liver Disease Research Center, Department of Medicine, College of Medicine, King Saud University, Riyadh, Saudi Arabia; 15 National Guard Hospital in Riyadh, Riyadh, Saudi Arabia; 16 Gastroenterology Unit, Department of Medicine, King Abdulaziz Medical City, Jeddah, Saudi Arabia; 17 Cho Ray Hospital, Ho Chi Minh City, Vietnam; 18 The BEAD Core (Biostatistics, Epidemiology, and Data Management), Johns Hopkins University School of Medicine, Baltimore, MD USA; 19 Department of Molecular and Clinical Cancer Medicine, Institute of Systems, Molecular and Integrative Biology, University of Liverpool, Liverpool, UK; 20 Division of Gastroenterology & Hepatology, Johns Hopkins University, Baltimore, MD USA; 21 Liver Transplant Center, King Faisal Specialist Hospital & Research Center, Riyadh, Saudi Arabia; 22 Center of Excellence for Liver Disease in Vietnam, Division of Gastroenterology & Hepatology, Department of Medicine, Johns Hopkins University School of Medicine, Baltimore, MD USA

**Keywords:** GALAD score, HCC early detection, Liver cancer, Vietnam, Saudi Arabia, Liver cirrhosis, Biorepository, Phase IV biomarker validation study

## Abstract

**Background:**

Vietnam and Saudi Arabia have high disease burden of primary hepatocellular carcinoma (HCC). Early detection in asymptomatic patients at risk for HCC is a strategy to improve survival outcomes in HCC management. GALAD score, a serum-based panel, has demonstrated promising clinical utility in HCC management. However, in order to ascertain its potential role in the surveillance of the early detection of HCC, GALAD needs to be validated prospectively for clinical surveillance of HCC (i.e., phase IV biomarker validation study). Thus, we propose to conduct a phase IV biomarker validation study to prospectively survey a cohort of patients with advanced fibrosis or compensated cirrhosis, irrespective of etiologies, using semi-annual abdominal ultrasound and GALAD score for five years.

**Methods:**

We plan to recruit a cohort of 1,600 patients, male or female, with advanced fibrosis or cirrhosis (i.e., F3 or F4) and MELD ≤ 15, in Vietnam and Saudi Arabia (*n* = 800 each). Individuals with a liver mass ≥ 1 cm in diameter, elevated alpha-fetoprotein (AFP) (≥ 9 ng/mL), and/or elevated GALAD score (≥ -0.63) will be scanned with dynamic contrast-enhanced magnetic resonance imaging (MRI), and a diagnosis of HCC will be made by Liver Imaging Reporting and Data System (LiRADS) assessment (LiRADS-5). Additionally, those who do not exhibit abnormal imaging findings, elevated AFP titer, and/or elevated GALAD score will obtain a dynamic contrast-enhanced MRI annually for five years to assess for HCC. Only MRI nearest to the time of GALAD score measurement, ultrasound and/or AFP evaluation will be included in the diagnostic validation analysis. MRI will be replaced with an abdominal computed tomography scan when MRI results are poor due to patient conditions such as movement etc. Gadolinium-ethoxybenzyl-diethylenetriamine pentaacetic acid-enhanced MRI will not be carried out in study sites in both countries. Bootstrap resampling technique will be used to account for repeated measures to estimate standard errors and confidence intervals. Additionally, we will use the Cox proportional hazards regression model with covariates tailored to the hypothesis under investigation for time-to-HCC data as predicted by time-varying biomarker data.

**Discussion:**

The present work will evaluate the performance of GALAD score in early detection of liver cancer. Furthermore, by leveraging the prospective cohort, we will establish a biorepository of longitudinally collected biospecimens from patients with advanced fibrosis or cirrhosis to be used as a reference set for future research in early detection of HCC in the two countries.

**Trial registration:**

Name of the registry: ClinicalTrials.gov

Registration date: 22 April 2022

Trial registration number: NCT05342350

URL of trial registry record

## Background

Vietnam and Saudi Arabia have some of the highest disease burdens of primary hepatocellular carcinoma (HCC) in the world [1]. More than 80% of HCC cases develop on the background of advanced fibrosis or cirrhosis [2]. Up to 90% of advanced fibrosis/cirrhosis cases, hence high risk for HCC, are due to hepatitis B virus (HBV), hepatitis C virus (HCV) and/or non-alcoholic steatohepatitis (NASH) in Vietnam or Saudi Arabia. The incidence of HCC in patients with HBV or HCV and/or NASH-associated advanced fibrosis is as high as 4% per year [3, 4], for cirrhosis 8% per year [2], making HBV-HCV or NASH in the presence of significant fibrosis the most significant risk factor for HCC development. Furthermore, up to 70% of newly diagnosed HCC cases in Vietnam and Saudi Arabia are at an advanced symptomatic stage, e.g., with Barcelona Clinic Liver Cancer staging system (BCLC)-C or D [5, 6]. Likewise, recent publications in Vietnam and Saudi Arabia documented that 80–92% of patients with HCC presented symptomatically [6, 7]. These data indicate that most patients with HCC in Vietnam or Saudi Arabia seek medical attention too late in their disease course; thus, therapeutic interventions are suboptimal at diagnosis.

Early detection in asymptomatic patients is a strategy to improve survival outcomes in HCC management. GALAD score is a serum biomarker-based panel that can improve early HCC detection in patients with chronic liver disease, including liver fibrosis and cirrhosis. In this protocol, GALAD will combine gender and age with the results from alpha-fetoprotein (AFP), lens culinaris agglutinin (LCA) bound fraction of AFP (AFP-L3%), and protein induced by vitamin K absence-II (PIVKA-II) or des-gamma-carboxy-prothrombin (DCP) levels. This score has been internally and externally validated [8] and recently received breakthrough designation from the United States Food and Drug Administration (US FDA). The performance of GALAD has been evaluated as a surveillance test for HCC in the United States, United Kingdom, Germany, Japan, and Hong Kong in case–control studies as well as in studies with design as PROBE (prospective specimen collection and retrospective blinded evaluation) [9]. However, it has not yet been evaluated in Vietnam or Saudi Arabia [8]. And most importantly, GALAD has not been investigated and validated prospectively for clinical surveillance of HCC in which GALAD score is applied to individuals in real-time and diagnostic procedures are performed for those with an elevated GALAD test (≥ -0.63).

In order to provide robust data for the potential use of GALAD in Vietnam and Saudi and as the next step in GALAD score biomarker development [10], we propose to conduct a phase IV biomarker validation study to prospectively survey a cohort of patients at risk for HCC (i.e., patients with advanced fibrosis or compensated cirrhosis and irrespective of cirrhosis etiologies), using semi-annual abdominal ultrasound and GALAD Score for five years. In doing so, we aim to validate the potential role of GALAD Score for clinical surveillance and early detection of HCC in Vietnam and Saudi Arabia. Additionally, we will collect and archive biospecimens to promote future research in chronic liver disease in Vietnam and Saudi Arabia.

## Methods / design

### Objectives

The primary objective of the study is to prospectively evaluate the performance of GALAD score as a biomarker-based surveillance model to detect early HCC in patients with advanced fibrosis or cirrhosis (e.g., Metavir F3 or higher, with model for end-stage liver disease [MELD] 15 or lower). The secondary objective is to collect and archive biospecimens to develop a bio-repository for future studies in chronic liver diseases.

### Outcome measures

The primary endpoint is the performance of GALAD score determined in association with HCC detection by LiRADS criteria in a cohort with advanced fibrosis or cirrhosis (e.g., Metavir F3 or higher, with MELD 15 or lower) undergoing prospective surveillance every six months for five years. Performance of GALAD score will be determined by using the GALAD score cut-off value of -0.63 to prospectively survey a cohort of 1,600 patients with advanced fibrosis and early cirrhosis (e.g., Metavir F3 or higher, with MELD 15 or lower) for early detection of HCC by Liver Imaging Reporting and Data System (LiRADS) assessment (LiRADS-5). The GALAD cut-off value was previously determined in case–control studies [11]. For the establishment of the biorepository, the endpoint is the proportion of study participants who agree to consent for bio-specimen when invited.

### Study design

This will be a prospective observational cohort phase IV biomarker validation study using semi-annual abdominal ultrasound and GALAD score.

### Study population

We plan to recruit a cohort of 1,600 patients, male or female, with advanced fibrosis/cirrhosis F3 or F4, and MELD ≤ 15, in Vietnam (*n* = 800) and Saudi Arabia (*n* = 800).

### Eligibility criteria

An individual must meet all the following criteria for inclusion in the study:

- Adults aged 18 or older
- All genders and ethnicities
- Diagnosis of fibrosis and cirrhosis based on histology and/or image showing cirrhotic liver with splenomegaly and platelet counts < 120 mm^3^, or esophageal or gastric varices on endoscopy AND presence of chronic liver disease/Fibroscan and/or Fib-4 and/or aspartate aminotransferase (AST) to platelet ratio index (APRI)/ acoustic radiation force impulse (ARFI). For viral hepatitis: transient elastography (TE) ≥ 9 kPa, APRI ≥ 1; for non-alcoholic fatty liver disease (NAFLD)/NASH: TE > 8 kPa, FIB-4 > 1.3
- Individuals already confirmed having cirrhosis with MELD ≤ 15 from any etiology (chronic HBV, chronic HCV, NASH, cirrhosis, etc.)
  - No significant hepatic decompensation
  - No hepatorenal syndrome
- For chronic HBV and/or HCV carrier, with or without treatment
  - No prior or current treatment for HCC
  - No cancer history within five years
  - No participation in a trial for HCC treatment
  - No prior solid organ transplant
  - Albumin, bilirubin, creatinine, and international normalized ratio (INR) labs within the past 30 days
  - AFP labs within 180 days irrespective of AFP titer
  - Imaging showing no HCC within 180 days
- For other medical history
  - No known AIDS-related diseases
  - No significant co-morbid conditions with life expectancy < 5 years
  - No other cancer(s)
- Agree to the collection of biosamples (serum, plasma, and urine) at each of the six months follow-ups during the study duration
- Provision of signed and dated informed consent form
- Stated willingness to comply with all study procedures and availability for the duration of the study and up to five years post-study follow up
- Willingness to give written informed consent to be enrolled in the database
- Resides in Vietnam or Saudi Arabia at the time of study and provides contact information (email and/or cell phone number for texting)
- Two phone numbers and personal identification numbers (citizen identification number)

A patient who meets any of the following criteria will be excluded from participation in this study:

- Decompensated cirrhosis (variceal bleeding, hepatic encephalopathy, ascites, spontaneous bacterial peritonitis, and/or hepatorenal syndrome) or MELD > 15
- Individuals who already have HCC, with or without HCC treatment
- On liver transplantation list or anticipated to be on the liver transplantation list during the study duration
- Any serious or active medical or psychiatric illness that, in the opinion of the investigator, would interfere with patient treatment, assessment, or compliance with the protocol
- Known HIV positive status
- Taking Vitamin K within seven days prior to clinic follow or having disease affecting Vitamin K levels.
- Active drug use or dependence that, in the opinion of the study investigator, would interfere with adherence to study requirements
- Inadequate documentation
- Individuals who cannot, do not want to, or refuse to sign the informed consent form

### Study settings

**Fig. 1:**
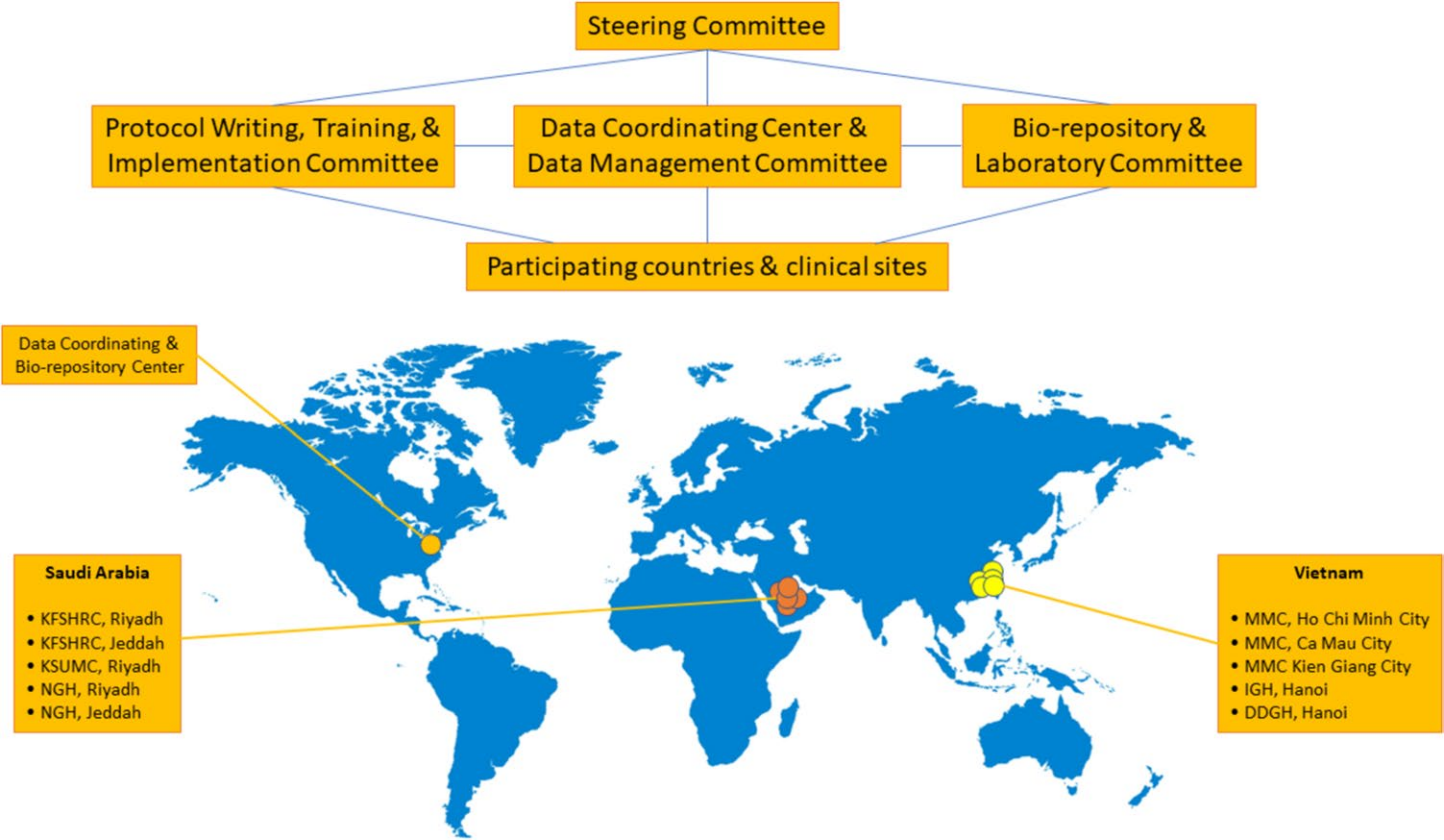
Team Organizational Chart and Study Sites. Steering *Committee*: Composed of the principal investigators, co-investigators, and consultants. Reviews the protocols and monitors the progress of the studies and participant safety. Ensures the research study is implemented in compliance with the protocol and that conflicts of interest and bias are minimized. Supported by sub-committees as illustrated. All protocols were approved by the Steering Committee and the Institutional Review Boards of the participating sites in Vietnam and Johns Hopkins School of Medicine, and all participants provided written informed consent. The clinical sites in Vietnam and Saudi Arabia are listed. Johns Hopkins School of Medicine serves as Data Coordinating Center. Abbreviations: DDGH-Dong Da General Hospital, IGH-Institute of Gastroenterology and Hepatology, KFSHRC-King Faisal Specialist Hospital and Research Center, KSUMC-King Saud University Medical City, MMC-Medic Medical Center, NGH-National Guard Hospital. Map Source: World map was taken from https://www.freepik.com/free-vector/illustration-of-global-icon_2687446.htm using a premium license (subscription ID: 9765e7de-0ed0-4358-b277-90cfe44e72a9) for unlimited use without attribution

Patients will be recruited at Hepatology Clinic, Viral Hepatitis Clinic, and/or Infectious Diseases Clinic of the following major hospitals in Vietnam and Saudi Arabia (Fig. 1):

- Medic Medical Center in Ho Chi Minh City, Vietnam
- Medic Medical Center in Ca Mau City, Vietnam
- Medic Medical Center in Rach Gia City, Vietnam
- Institute of Gastroenterology and Hepatology, Hanoi, Vietnam
- Dong Da General Hospital, Hanoi, Vietnam
- King Faisal Specialist Hospital and Research Center, Riyadh, Saudi Arabia
- King Faisal Specialist Hospital and Research Center, Jeddah, Saudi Arabia
- King Saud University Medical City, Riyadh, Saudi Arabia
- National Guard Hospital, Riyadh, Saudi Arabia
- National Guard Hospital, Jeddah, Saudi Arabia

### Study schedule and assessments

**Fig. 2:**
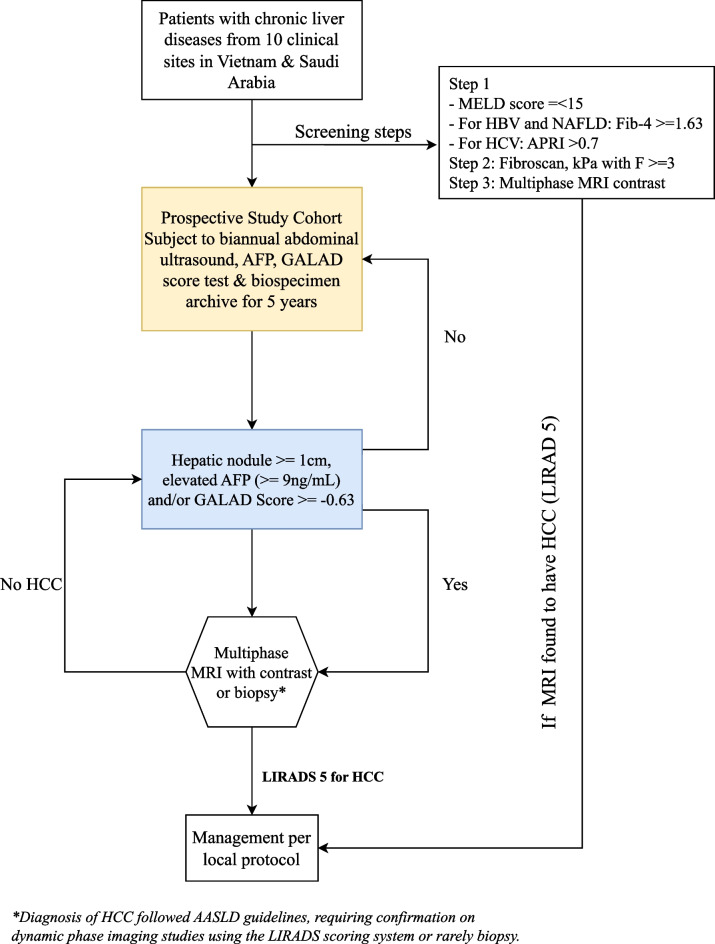
Study Schema. Patients with chronic liver diseases from hospital database of the participating sites will be screened and invited for enrollment if eligible. Next, we prospectively survey the eligible cohort of patients at risk for HCC (i.e. patients with advanced fibrosis or compensated cirrhosis and irrespective of cirrhosis etiologies), using semi-annual abdominal ultrasound and GALAD Score for five years. During the 5-year follow-up, individuals with a liver mass ≥ 1 cm in diameter and/or elevated GALAD score (≥ -0.63) will be scanned with dynamic contrast-enhanced MR,I and a diagnosis of HCC will be made by LiRADS assessment (LiRADS-5). Additionally, those who do not exhibit abnormal imaging findings and/or elevated GALAD score will obtain a dynamic contrast-enhanced MRI annually for five years to assess for HCC

Figure 2 depicts the study schema. The study is planned for five years. Individuals with a liver mass ≥ 1 cm in diameter, elevated AFP (≥ 9 ng/mL), and/or elevated GALAD score (≥ -0.63) will be scanned with dynamic contrast-enhanced MRI, and a diagnosis of HCC will be made by LiRADS-5. Additionally, those who do not exhibit abnormal imaging findings, elevated AFP titer, and/or elevated GALAD score will obtain a dynamic contrast-enhanced MRI annually for five years to assess for HCC. In Vietnam, the study sites and the funds to be received for the study will be used for conducting all the tests and MRIs. National health insurance will not be used. In Saudi Arabia, national health insurance will cover the participants’ tests and MRIs.

During the 5-year time frame, we anticipate at least 80 (approximately 16 each year if 1% incidence x five years for both cohorts in Vietnam and Saudi Arabia) or as many as 320 (64 per year if 4% incidence x five years for both cohorts in Vietnam and Saudi Arabia) incident HCC cases given the high HCC incidence rate in Vietnam and Saudi Arabia. With the range of 80 to 320 anticipated HCC cases, we will have 90% to 99% power, respectively, to detect a meaningful difference in the cases vs. control (more on sample size calculation below). We will compare sensitivity for early HCC detection (primary outcome measures), false-positive results, and resultant diagnostic evaluation.

By leveraging the prospective cohort, we will establish a biorepository of longitudinally collected biospecimens from patients with fibrosis/cirrhosis to be used as a reference set for future research. Clinical, laboratory, and imaging data, urine, and plasma biospecimens are from participants for up to five years; data are collected only from participants who maintain the absence of evidence of HCC on imaging and/or biopsy. Biospecimens and associated de-identified clinical data from participants enrolled in this study will be stored in Vietnam for participants from Vietnam and in Saudi Arabia for participants enrolled from Saudi Arabia.

### Screening

Before or during the first visit, patients with chronic liver diseases, including advanced fibrosis/cirrhosis identified by ICD-10 from participating clinical sites, will be screened.

**Fig. 3:**
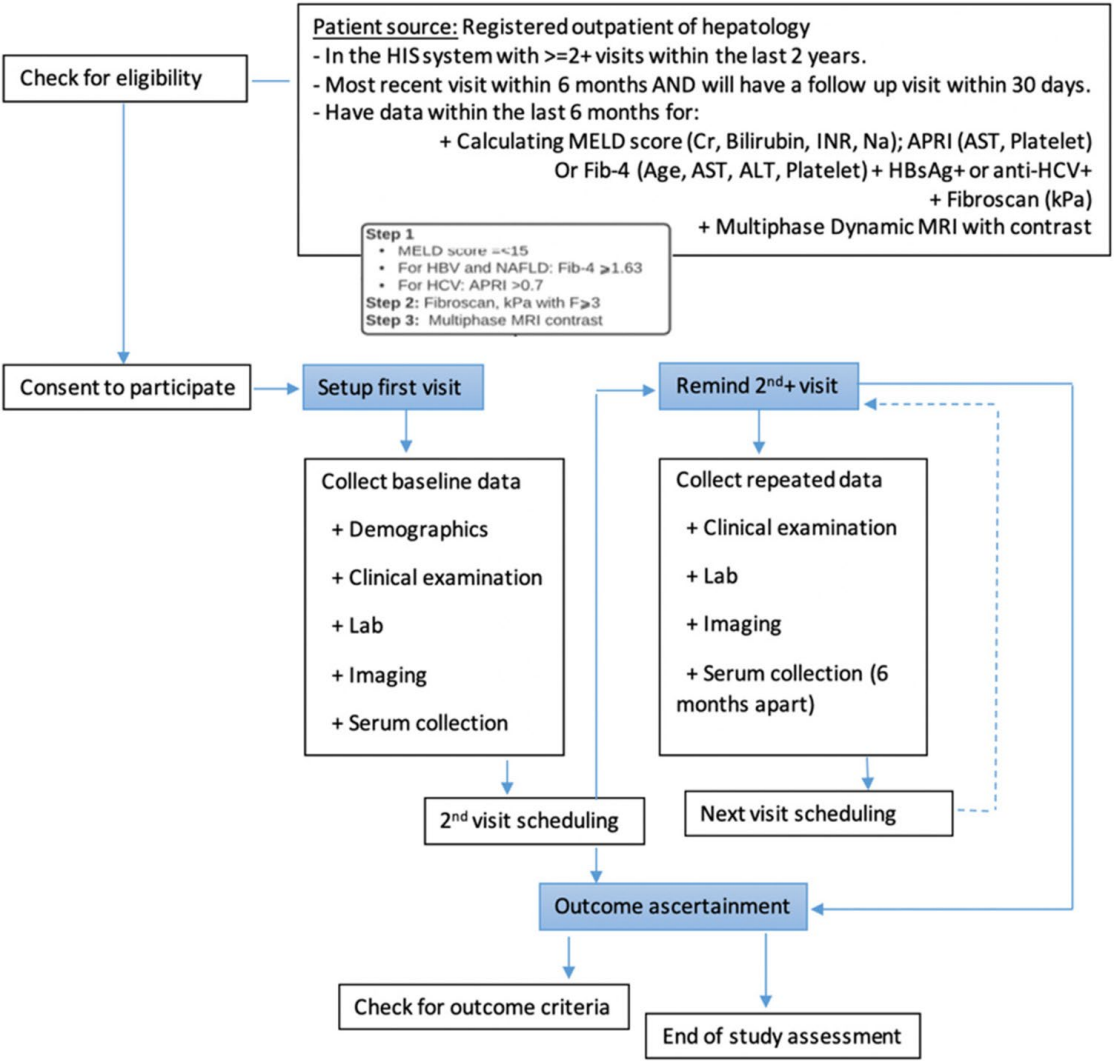
Overall Patient Screening and Enrollment Flow. The study will be introduced at the 10 sites for the outpatient internal, hepatology, gastrointestinal, infectious diseases doctors, and viral hepatitis management team to ensure all the doctors approaching the target population know about the study. Prospective patients were screened for eligibility for the inclusion criteria and exclusion criteria by the study coordinators. Patients who fulfilled inclusion criteria will be introduced and explained about the study by the study coordinators and or site principal investigators. At the initial screening visit, the details of the study will be introduced. If the participant is agreeable and thought to have met the inclusion and exclusion criteria, then he/she may enter the formal study enrollment phase. The signing of the consent statement and the procedures during screening and enrollment can occur on one day or separate calendar days and may occur over a period of up to 30 days. Subsequent study follow-ups and procedures are shown

The screening criteria are MELD ≤ 15, non-invasive indices, and finally confirmed by Fibroscan or ARFI elastography results to further select those with fibrosis score F3 or higher from the 10 clinical centers’ databases in this Study Cohort Database (Fig. 1 & 3). All patients will be required to have a negative dynamic contrast-enhanced MRI upon study enrollment to rule out HCC before the study enrollment. Only MRI nearest to the time of GALAD score or abdominal ultrasound (US) and or AFP evaluation will be included in the analysis. If patients have a history of metal in their heads or eyes, they will need an x-ray of their skull to find out if the MRI is safe for them.

### Enrollment

The participant is considered enrolled in the study once the consent is signed and the Registration Form has been completed. Activities at the initial screening and or study registration visit include:

- Sign the forms
- Signature on the study consent and biorepository consent form
- Signature on the Health Insurance Portability and Accountability Act (HIPAA) authorization form for the study
- Assignment of the study participant identification number
- Medical and medication history
- Physical examination including vital signs, height, weight, anthropometric measurements, handgrip strength measurement
- Screening electrocardiogram (ECG)
- Acanthosis nigricans and liver signs
- Obtaining laboratory and other information (on the co-variates list)
- Instructing participant to bring to initial screening visit his/her health history information or related materials
- Participant to sign medical records release to obtain study labs and imaging
- Participant to provide location and contact information
- Coordinator to register participants on clinic data system
- Coordinator to request prior reports and study imaging/procedures from healthcare provider
- Laboratory test
  - Hematology (complete blood count)
  - Chemistry (hepatic panel, hemoglobin A1c (HbA1c), fasting lipid profile and fasting glucose and insulin levels, fasting blood (plasma) for specimen banking
- Imaging diagnostic: abdominal ultrasound, Fibroscan, or ARFI
- Liver biopsy (if needed)
- Provision of the standard of care educational materials (delay providing these to the participant until confirmed eligible for the study)
- Schedule for the second visit

### Participant retention & compensation

We will disseminate flyers comprising vital study information in hepatology clinics of the study sites. We also have databases of prospective study candidates at the study sites. Our strategy to recruit and follow-up the study patients to minimize the drop-out is by emphasizing the following:

1. selecting the committed candidates by checking on their response time following the invitation and implementing an ask-back method to check for their understanding of the study,
2. setting up patient expectations and roadmap when they join the study,
3. individually reminding and scheduling patient follow-up appointments using automation systems,
4. incentivizing study patients with parking lot costs (or transportation vouchers) and study tests/MRIs during the studies (all these incentives have been budgeted),
5. sending ‘thank you’ notes to study patients after each visit to enhance the trusting relationship between the study staff and study patients.

The participants will have free laboratory tests and MRI annually. They will also be compensated for the transportation costs for 10 visits.

### Schedule of activities

Data will be collected during screening (initial visit) and at semi-annual intervals thereafter (a maximum of ten visits). Schedule of activities (Table 1) displays the data collection schedule for screening and follow-up.

**Table 1:** Schedule of activities

Procedures	Screening/baseline (Visit 1)	Study Visit 2	Study Visit 3	Study Visit 4	Study Visit 5	Study Visit 6	Study Visit 7	Study Visit 8	Study Visit 9	Final Study Visit 10
Informed consent	X									
Demographics	X									
Home address/Phone number	X	X	X	X	X	X	X	X	X	X
Medical history	X	X	X	X	X	X	X	X	X	X
Collect and archive serum (15 mL blood, 50 mL urine)	X	X	X	X	X	X	X	X	X	X
Concomitant medication review	X	X	X	X	X	X	X	X	X	X
Physical exam (including height and weight)	X	X	X	X	X	X	X	X	X	X
Vital signs	X	X	X	X	X	X	X	X	X	X
Height	X									
Weight	X	X	X	X	X	X	X	X	X	X
Waist circumference	X	X	X	X	X	X	X	X	X	X
Performance status	X	X	X	X	X	X	X	X	X	X
Handgrip strength measurements	X	X	X	X	X	X	X	X	X	X
Hematology	X	X	X	X	X	X	X	X	X	X
Serum chemistry	X	X	X	X	X	X	X	X	X	X
Adverse event review and evaluation		X	X	X	X	X	X	X	X	X
Radiologic/Imaging assessment (Abdominal Ultrasound and/or cross section imaging if triggered)	X	X	X	X	X	X	X	X	X	X
Dynamic contrast-enhanced CT or MRI	X		X		X		X			X
Fibroscan	X									X
Complete Case Report Forms (CRFs) that have clinical data and events	X	X	X	X	X	X	X	X	X	X

### Follow-up visits

Semi-annual follow-up visits will be scheduled at 22 to 26-week intervals after enrollment. Each visit has an ideal date for a visit, a lower window date (opening date for the window), and an upper window date (closing date for the window). The dates for a specific participant are specified on their visit time windows sheet. In addition to the activities in Table 1, new procedures and forms are to be completed at each of the follow-up visits (at 6, 12, 18, 24, 30, 36, 42, 48, and 54 months) are:

- Follow-up medical history (medication changes, key events or interventions, surgeries)
- Hospital admissions, new diagnoses of co-morbidities, complications of liver disease (variceal bleeding, ascites, edema, hepatic encephalopathy), liver cancer, other cancer, diabetes
- Physical examination
- Abdominal US and AFP
- Laboratory data (hematology, glucose, insulin, clinical chemistry, hepatic panel, HbA1c, lipid profile)
- Blood collection for plasma banking
- Documentation of any additional liver biopsies performed

Additionally, annually, each study participant will obtain a dynamic MRI with contrast to evaluate for liver cancer.

### GALAD score

This study is considered a prospective observational cohort study. GALAD score, comprising Gender, Age, AFP-L3, AFP, and DCP, is an add-on test to the routine HCC surveillance care with the provisions of abdominal US and AFP every six months.

Japanese investigators have, for several decades, combined AFP with two additional markers, DCP and AFP-L3, for diagnosis and surveillance. DCP, also known as Protein Induced by Vitamin K Absence or Antagonist-II (PIVKA-II) is an immature form of prothrombin [12, 13]. Elevated DCP values (≥ 7.5 ng/ml) are associated with a fivefold increased risk of developing HCC, and on this basis, DCP has received USFDA approval for risk assessment. AFP-L3, a glycoprotein normally produced by the fetal liver, is one of three AFP glycoforms that can be separated based on their lectin binding characteristics, most readily with LCA. An increase in AFP-L3 appears more specific for HCC than total AFP in adults. It is usually presented as a percentage of the total AFP with a reference range of < 10%.

GALAD formally combines these three serum biomarkers together with age and gender to produce an algorithm with better performance than its individual constituents. The GALAD model is of the form: Z = -10.08 + 0.09 × age + 1.67 × sex + 2.34 log (AFP) + 0.04 × AFP-l3 + 1.33 × log (DCP). Where sex = 1 for males, 0 for females.

### Variables assessments and definition

Upon study entry, each participant will have the following lab tests and imaging study:

- MELD score ≤ 15 (regardless of etiology) based on INR, total bilirubin, serum creatinine of latest test results from the most recent visit within six months of entry or at entry
- Child–Pugh score grade A (optional, if available)
- APRI > 0.7 in patient with anti-HCV positive based on anti-HCV (no time limit), AST, alanine aminotransferase (ALT), and platelet count
- Fib-4 index ≥ 1 in patients who are hepatitis B surface antigen (HBsAg) positive or those with NAFLD based on HBsAg, AST, ALT, platelet count, and age
- Fibroscan kPa (HBV etiology is kPa ≥ 9, for HCV kPa ≥ 9, and for NAFLD kPa ≥ 8)
- Other variables from case report form (CRF): “Yes” vs. “No” for dichotomous variable
- Dynamic contrast-enhanced MRI to rule out HCC
- GALAD score based on Gender, Age, AFP, AFP-L3, and PIVKA-II (DCP) of latest test results from the most recent visit within six months of entry.

### Outcome ascertainment and study exit definitions

- Time of positive surveillance: latest date of visit with lab results
- Criteria for outcome assessment: elevated GALAD score and/or suspicion of liver tumor on ultrasound, whichever comes first
- Date of detection of liver tumor ≥ 1 cm in size on dynamic contrast-enhanced MRI or confirmation of HCC on biopsy
- Outcome captured if multiphase MRI with contrast is not needed when GALAD score is less than the cut-off and ultrasound findings are normal.
- Tumor of any size and quantity will be recorded.
- Person-years of follow-up time: calculated for each participant from one year after the enrollment date to the event date (indication for multiphase MRI with contrast; HCC diagnosis; date of loss to follow-up, or end of study, whichever comes first).
- Time to event: from entry in the study to the date of liver tumor detection on multiphase MRI with contrast or on the date of biopsy with HCC confirmation.
- GALAD score and ultrasound: within four weeks of the visit with indication
- Loss to follow-up: 12 months of last clinic encounter with no study visits
- End of study: completion of the last visit or procedure shown in Table 1 or when a patient develops HCC and then subject to the local management protocol (above)

### Data collection and data management

Demographic details (age, gender, race/ethnicity, marital status, district/county of residence, and insurance), clinical data (CRFs), laboratory data (whole blood count, comprehensive metabolic panel, INR, fasting lipid panel, HbA1C, and AFP), imaging details (ultrasound, Fibroscan [or ARFI], MRI, ECG), biospecimen for storage (plasma, urine, and if feasible, tissue), and other data specified in Table 1 will be collected from each patient.

Patient-reported data will be extracted from the hospital information systems or collected based on a structured form and will be uploaded to a central database using a pre-formatted data structure. Experienced, skilled, locally licensed radiologists from the study sites in Vietnam and Saudi Arabia will read the MRI results and interpret the findings. Additionally, an independent radiologist with similar practicing experience will re-read the MRI and validate the interpretations. Trained and experienced research staff will handle data collection and management. Data will be stored in a secure setting to maintain privacy. The data monitoring committee will be comprised of the Principal Investigators, Data Management Co-Investigator, Overall Study Manager and Vietnam Study Manager, Saudi Arabia Study Manager, and Biostatistician.

### Missing data

The missing data and data of those who lost to follow-up will be analyzed and reported in two ways. One is that we will treat them as incomplete data, remove them from the analyses, and only report the results of those who have completed data. The second way is to impute the data as complete cases based on other cases with complete data, subject them to sensitivity analysis, and subsequently report them as complete data.

When we calculated the sample size for the study, we accounted for up to 30% missing data or loss to follow up.

### Safety reporting

Unanticipated or adverse events will be monitored and reported to ensure participant safety. Serious adverse events will be reported upon discovery at the clinical center. Participants returning to clinic at different time point(s) not pre-determined; those found to have elevated AFP or hepatic nodules that are not during the study visits; and other events during patient follow-up, i.e. cirrhosis decompensation, incidental findings of hepatic mass detected not by the study, clinic presentation outside of study follow-up date ranges, inconclusive findings of hepatic nodule by imaging and liver biopsy will be monitored, reviewed, and reported.

### Sample size

Two estimates were calculated for the sample size, assuming low (1%) and high (4%) annual incidence rates in the population with the highest risk for HCC. Assuming an annual incidence of 1%, 90% power and alpha of 0.05 during the 5-year follow-up period, we anticipate a 5-year incidence proportion of 0.05 (5*0.01). The sample size needed is 621 (each country). For a 4% annual incidence rate using the same assumptions, the sample size required is 119. To err on the conservative side, we aimed to recruit 800 per country (Vietnam and Saudi Arabia) to account for the loss to follow-up. These sample size calculations were performed in Stata 15.1 using the “sampsi” command.

### Statistical methods

Bootstrap resampling technique will be used to account for repeated measures to estimate standard errors and confidence intervals. Only MRI nearest to the time of GALAD, US and/or AFP evaluation will be included in the analysis. For time-to-event data, we will use the Cox proportional hazards regression model with covariates tailored to the hypothesis under investigation. For hypotheses involving repeated measurements, events, counts, or other discrete responses, we will use either of two approaches: (1) generalized linear models with generalized estimating equations with robust variance estimation to account for the clustering, or (2) multilevel generalized linear mixed models with random coefficients to account for within-patient clustering as well as other sources of variations like clinic effects.

We will include a random intercept for each hospital site to account for variability by facility. Stratified analyses will be presented for p for interaction < 0.2. Demographic and clinical characteristics will be summarized with proportions and median (minimum–maximum) with no calculation of p-values as this is a non-randomized study. Age at liver disease diagnosis and sex will be accounted for in all analyses. Other confounders of interest include interval follow-up and insurance coverage, whereas age and gender are already controlled in the GALAD (gender, age, etc.) score calculations. All statistical analyses will be performed in SAS 9.4 (or R or another statistical software package with equivalent capabilities).

Baseline descriptions of patients with the standard of care (US and AFP) vs. GALAD score will be compared to each other. Other baseline demographics, biochemical lab results, MRI results, Fibroscan, and abdominal US in the cohort will also be described.

The interim analyses will re-evaluate the performance of the GALAD cut-off score of -0.63 after year 2 of the study. The interim analyses will not be used to assess for study events (i.e., HCC cases), and thus there will be no plan to stop the study if there were no number of events (i.e. HCC cases).

### Authorship

The authorship eligibility guidelines by the International Committee of Medical Journal Editors (https://www.icmje.org/recommendations/browse/roles-and-responsibilities/defining-the-role-of-authors-and-contributors.html) will be followed to determine the authorship contribution.

## Discussion

Assays of AFP, AFP-L3%, and/or DCP have been available for clinical use in a few medical centers in Vietnam over the past five years [14]. Locally, the main indication for the use of the assay is to aid in the risk stratification for HCC in the cases of hepatic nodules with atypical features on cross-sectional imaging for HCC diagnosis, such as without arterial phase hyperenhancement or without delayed phase washout appearance [14]. Other indication, in Vietnam at least, is when AFP has risen upper limits of normal (locally > 9 ng/mL) [14], which often occurs in advanced HCC. There are no consensus national practice guidelines for the use of biomarkers [14]. Similar to Vietnam, GALAD score uptake in Saudi Arabia has not been widely used in clinical practice. Reasons for this include the lack of validated prospective data on GALAD score [6].

Taken together, the combined GALAD score has not been validated and thus utilized systematically for HCC surveillance in Vietnam or Saudi Arabia. Additionally, there is a lack of wide and uniform uptake of GALAD in clinical practice in Vietnam and Saudi Arabia, and there is a need for high-quality research on the use of the GALAD for HCC surveillance. The present work will attempt to fill this evidence gap by evaluating the performance of the GALAD score in early detection of liver cancer. Furthermore, by leveraging the prospective cohort, we will establish a biorepository of longitudinally collected biospecimens from patients with advanced fibrosis or cirrhosis to be used as a reference set for future research in the two countries.

### Confidentiality

All laboratory specimens, evaluation forms, reports, and other records that are part of the study data collection and entry materials will be identified by coded number only to maintain subject confidentiality. All records will be kept in locked file cabinets with access limited to the study investigators. All computer entry and networking programs will identify subjects by participant identification number. Clinical information will not be released without written permission of the subject, except as necessary for monitoring by the IRB or data safety monitoring board. Clinical information may be reviewed during site visits, but the use of personal identifiers will be avoided. Consent procedures and forms as well as the communication, transmission, and storage of participant data will comply with individual site IRB and HIPAA.

## Data Availability

The datasets that will be generated and/or analyzed during the study (to be conducted using this protocol) will be made available appropriately. Corresponding authors may be contacted if someone wants to request the data from this study.

## Abbreviations

AFP: Alpha-fetoprotein
AFP-L3%: Lens culinaris agglutinin bound fraction of AFP
ALT: Alanine aminotransferase
APRI: Aspartate aminotransferase (AST) to platelet ratio index
ARFI: Acoustic radiation force impulse
AST: Aspartate aminotransferase
BCLC: Barcelona Clinic Liver Cancer staging system
CRF: Case report form
DCP: Des-gamma-carboxy-prothrombin
FIB-4: Fibrosis-4 score
GALAD: Gender, Age, AFP-L3%, AFP, and DCP
HBsAg: Hepatitis B surface antigen
HBV: Hepatitis B virus
HCC: Hepatocellular carcinoma
HCV: Hepatitis C virus
HIPAA: Health Insurance Portability and Accountability Act
INR: International normalized ratio
IRB: Institutional review board
LCA: Lens culinaris agglutinin
LiRADS: Liver Imaging Reporting and Data System
MELD: Model for end-stage liver disese
MRI: Magnetic resonance imaging
NASH: Non-alcoholic steatohepatitis
PROBE: Prospective specimen collection and retrospective blinded evaluation
PIVKA-II: Protein induced by vitamin K absence-II
TE: Transient elastography
US: Ultrasound
